# Traumatic Atrial Septal Defect Repair via Primary Endovascular Suture Method: Case Report and Operative Technique

**DOI:** 10.7759/cureus.33679

**Published:** 2023-01-12

**Authors:** Sandor Toledo, Christopher Wang, Megan Satyadi, Papus Keita, Anthony Nobles, Timothy Misselbeck, Bryan Kluck

**Affiliations:** 1 Surgery, Lehigh Valley Hospital Network, Allentown, USA; 2 Surgery, University of South Florida Morsani College of Medicine, Tampa, USA; 3 Surgery, Carle Foundation Hospital, Urbana, USA; 4 Biomedical Engineering, HeartStitch, Inc, Fountain Valley, USA; 5 Biomedical Engineering, West Sachsen University, Zwickau, DEU; 6 Cardiac Surgery, Lehigh Valley Hospital Network, Allentown, USA; 7 Cardiology, Lehigh Valley Hospital Network, Allentown, USA

**Keywords:** septal defect primary repair, intracadiac echocardiogram, traumatic septal defects, interventional cardiologist, atrial septal defects

## Abstract

We report a case of a 20-year-old male with no prior medical history who was found to have an atrial septal defect on echocardiography following a motor vehicle accident (MVA). The patient underwent primary percutaneous defect closure using the NobleStitch EL (Heartstitch, Fountain Valley, California) cardiovascular suturing system with intra-operative Doppler echocardiogram showing no residual shunt or color flow. There were no operative complications. At five months follow-up, the patient reported no symptoms from the procedure. In the case of traumatic atrial septal defect repair, the NobleStitch EL system may be utilized as an alternative to open heart surgery.

## Introduction

Traumatic atrial septal defects (ASDs) can be a rare sequela of blunt force trauma to the chest wall. The incidence of asymptomatic versus symptomatic cardiac septal defects following trauma is not well studied. In this case, a transesophageal echocardiogram (TEE) showed a blunt traumatic inter-atrial rupture with a rim of freely mobile tissue. The non-uniformity of the defect and the mobile tissue were thought to be more consistent with a traumatic ASD than a congenital ASD. There were concerns for improper seating of traditional metal patent foramen ovale (PFO) and septal occluder endovascular repair devices given the rim of freely mobile tissue. The patient underwent a primary endovascular suture repair using The NobleStitch EL system with an intra-operative Doppler echocardiogram showing no residual shunt or color flow. 

## Case presentation

Management

The patient presented to our emergency department following a significant unrestrained motor vehicle accident (MVA). He was initially found ejected from his vehicle and with altered mental status. Computed tomography (CT) imaging revealed multiple significant injuries involving the head, spine, and abdominal organs. A cardiac contusion was initially evaluated with an electrocardiogram and then a transthoracic echocardiogram (TTE), which showed a left-to-right shunt through the interatrial septum with concerns for congenital ASD versus blunt traumatic rupture (Video 1). 

**Video 1:** Transthoracic Echocardiogram (TTE) TTE showing left-to-right shunt through interatrial septum with concerns for congenital atrial septal defect (ASD) versus traumatic rupture.

Evaluation via transesophageal echocardiogram (TEE) showed a persistent ASD measuring 1.3 cm in maximum dimension with left-to-right shunting (Qp/Qs = 1.57) and an 0.8 cm rim of surrounding freely mobile tissue (Video 2). The right atrium and ventricle were mildly dilated with normal systolic function.

**Video 2:** Transesophageal Echocardiogram (TEE) TEE showing blunt traumatic ASD measuring 1.3 cm in maximum dimension with left-to-right shunting and an 0.8 cm rim of surrounding freely mobile tissue.

His injuries were stabilized over a month, and he was discharged home with home services. Assessment by our cardiac and cardiothoracic teams decided that a repair was indicated given the left-to-right shunt and right ventricular dilation. A less invasive approach via percutaneous repair was discussed during a multidisciplinary meeting and preferred by the patient. Special consideration was given to incorporating the freely mobile rim of tissue in the primary closure as it might allow for a better seal in the context of a non-uniform defect. Cardiothoracic surgery was on board with plans to proceed directly to the operating room if percutaneous suture repair was not successful. 

The patient proved to have issues with compliance after inpatient discharge by having a history of missed appointments and medication nonadherence. The NobleStitch EL and KwiKnot (Heartstitch, Fountain Valley, California) system utilize a percutaneous suture delivery apparatus to repair cardiac defects. Two propylene sutures, threaded through the opposing margins of the atrial septum to approximate the tissue and close the defect without leaving behind any apparatus or foreign bodies. Given the patient’s history, it was felt that loss of follow-up would have more somber complications in cases of embolization or erosion sometimes seen with larger devices. Furthermore, it was considered the safer choice given medication nonadherence since the system only requires short-term post-surgical antiplatelet coverage and no anticoagulation. He underwent successful blunt traumatic ASD repair six months after his initial injuries and was discharged home on three months of rivaroxaban and long-term daily aspirin. 

Follow-up

At five months follow-up, the patient reported no cardiac symptoms and was only taking aspirin.

Procedural technique

Bilateral ultrasound-guided femoral vein access was accomplished, and anticoagulation was administered to achieve an activated clotting time (ACT) of 250 seconds. Long 8 French sheaths were introduced into each of the common femoral veins using ultrasound guidance. The intra cardiac echocardiography (ICE) catheter was introduced via the left 8 French sheaths and a heart survey identified the primary ASD with no secondary defects. A 6 French multipurpose catheter and Amplatzer extra-stiff wire were placed through the right groin crossing the septal defect and into the left upper pulmonary vein (LUPV). This platform was used to exchange the right-sided sheath for a long 14 French sheath. The 0.035 wire was then exchanged for an 0.032 wire and placed into the LUPV.

A 25 mm sizing balloon was advanced over the 0.032 wire and used to map the ASD by collocating the nadir of the septum secundum and the superior extent of the septum to spinal bony landmarks at steep left anterior oblique (LAO) angulation. Of note, the sizing balloon is not used for attaining the exact size of the defect and thus it is not inflated anywhere near the required pressures for stop flow levels. Consequently, the balloon is gently inflated allowing the ASD to deform it, and poses little to no risk of further septal damage. Once gently inflated, the superior most indentation of the balloon defines the superior most aspect of the septum primum, then a contrast injection delivered through the sheath outlines the septum secundum. These two landmarks allow for the co-location of the suture catheters ensuring optimal suture delivery. Once sizing was completed, the balloon was removed and an additional 0.018 wire was placed through the right 14 French sheaths and advanced to the level of superior vena cava. At this time, the NobleStitch EL secundum device was introduced over both the 0.032 wire and the 0.018 wire and brought to the level of the interatrial septum. After achieving contact with the septum, the septum secundum suture was deployed and the NobleStitch EL secundum device was slightly retracted. The lever arm was closed, and the suturing catheter was retracted back into the sheath. The spool was inspected to confirm delivery of the suture and the 0.018 wire was removed (Video 3). 

**Video 3:** Heart catheterization and atrial septal defect (ASD) repair 1. 25 mm sizing balloon across the ASD at 60 degrees left anterior oblique (LAO) view. Negative contrast outline of the septum secundum. 2. Secundum catheter over both the 0.018 and 0.032 wires engaging the septum secundum and the suture deployment. 3. Primum catheter through the ASD over only the 0.032 wire and the primum suture deployment. 4. Post Quickknot deployment and release showing no significant contrast through the ASD.

The septum primum suture catheter was then loaded onto the remaining 0.032 wire and advanced into the left atrium. The device was carefully placed above the previously collocated margin of the defect and deployed into the septum primum. The septum primum catheter was advanced, and the lever closed. Again, positive suture retraction was applied by moving the suture within the spool. The 0.032 wire was then removed. At this time, the KwiKnot catheter was loaded onto the four exteriorized suture tails outside the right femoral vein access point. The KwiKnot device was advanced to the septal position, with placement confirmed by angiography. The KwiKnot was deployed, and the catheter was removed (Video 4). 

**Video 4:** Intracardiac echocardiogram (ICE) 1. 0.035 wire across the atrial septal defect (ASD) and into left upper pulmonary vein (LUPV). 2. Inflated sizing balloon across the ASD demonstrating "stop flow". 3. ICE image of the sutured ASD. Note the outline of the Quikknot in position against the right side of the closed septum.

Final angiography demonstrated satisfactory placement and ICE demonstrated no color flow through the septum (Video 5).

**Video 5:** Post-atrial septal defect repair color flow echocardiogram Post-atrial septal defect closure color flow echocardiogram demonstrating no appreciable flow across the atrial septum.

All sheaths were then removed, and the access sites were closed. A total of 150 mL of contrast and 1477 mGy of fluoroscopy were utilized for the procedure (Video 6). 

**Video 6:** Post-atrial septal defect repair bubble study Post-atrial septal defect closure agitated saline bubble study showing proper closure.

## Discussion

Atrial septal defects (ASDs) represent the most common cardiac abnormality in adults, accounting for 7 - 10% of congenital heart disorders [1]. The incidence of atrial septum defects in adults resulting from trauma has not been well reported. However, they have recognized complications of blunt force trauma to the chest wall [2]. In the traumatic setting, Ventricular Septal Defects (VSDs) are more common, especially in the penetrating classification [3]. An ASD caused by blunt trauma may present differently, as in this case. ASD’s that are congenital in nature commonly involve inadequate septal tissue development, resorption, or fusion. Here, freely mobile tissue at a point caudal to the septal defect comprised a rim surrounding the ruptured septum, suggesting a traumatic nature. Disrupted tissue presents a unique challenge for percutaneous correction, as traditional metal patent foramen ovale (PFO) and septal occluder devices may not seat properly. The NobleStitch EL system was chosen for this case because it does not require device placement into the defect. It was felt that a suture-based closure might allow for a better seal in the context of a non-uniform defect. Furthermore, since there is no residual hardware left behind, there is no need for post-operative anticoagulation and only a short-term course of antiplatelet medication. The NobleStitch EL received Food and Drug Administration clearance in the United States in 2017 for cardiovascular suturing, and CE Mark for PFO closure in Europe in 2012. It has primarily been used for PFO closures. 

Metal-based closure devices are well-established in the literature for the closure of traumatic ASDs and VSDs, with variable outcomes and complications resulting from the procedure or device. Device migration, embolization, residual shunting, tissue erosion, nickel allergy, and cardiac perforation are rare but reported complications of such devices [4, 5]. Successful ASD closure with permanent metal devices requires a precise selection of the device; selecting a device that is too large or too small may increase the risk of complications or lead to a failed closure. In some cases, particular device characteristics are more favorable based on septal anatomy or patient characteristics. 

The NobleStitch EL represents an alternative percutaneous closure procedure that overcomes some of the challenges surrounding septal device placement and allows for better closure in the context of anatomic disruption from trauma. Several cases have been reported demonstrating the utility of the NobleStitch in correcting residual symptomatic shunting following permanent metal device placement [6,7,8]. Utilization of the NobleStitch reduces the impact of confounding factors that influence device selection, such as the width and length of the defect, the integrity of the septal rims, and tissue compliance, all of which contribute to device failure. The first registry comprising results of percutaneous suture-mediated PFO closure technique using the NobleStitch EL had a 96% (186 patients) success rate with a 90% closure rate defined as grade 0 or 1 (non-significant right-to-left shunt) and no device-relation complications [8]. 

Possible NobleStitch EL complications include residual shunts following suture deployment given the complexity of some defects. Although uncommon, prompt identification on angiography allows for the delivery of an additional suture, not dissimilar to traditional surgical suture techniques. Patient selection is key and optimal operator technique is fundamental to placing the septum primum and secundum sutures. There has been a reported suture septal tear and iatrogenic ASD resulting in persistent left-to-right shunting that was treated with an Amplatzer Septal Occluder [6]. However, it is unclear what parameters favored alternate device utilization over the placement of a second suture.

Responsibility for the use of a product for an indication other than that previously approved on the label falls on the physician. The decision to use such a product requires the physician to be well informed on its limitations and be proficient in its use. Utilization of a marketed product in the “practice of medicine” that is based on firm scientific rationale and sound medical evidence does not require the submission of an Investigational New Drug Application (IND), Investigational Device Exemption (IDE), or review by an Institutional Review Board (IRB). Local IRB oversight was not required by the sponsoring institution.

## Conclusions

We report the first successful percutaneous closure of a traumatic ASD following blunt thoracic trauma with the NobleStitch EL cardiovascular suturing system in the United States. While percutaneous device implantation or surgical repair has been the mainstay for the repair of traumatic ASDs, the NobleStitch EL percutaneous suture system may be a superior minimally invasive, and effective alternative.
